# Muscle Metabolomic Responses of Dumont and Mongolian Sheep to Alfalfa Hay- and Corn Straw-Based Diets: An Untargeted Metabolomics Study

**DOI:** 10.3390/ani16101487

**Published:** 2026-05-12

**Authors:** Ran Zhang, Meila Na, Wenliang Guo, Jing Zhang, Renhua Na

**Affiliations:** 1 College of Animal Science, Inner Mongolia Agricultural University, Hohhot 010018, China; ran893287@163.com (R.Z.); 15548710467@163.com (M.N.); zhangjing230518@163.com (J.Z.); 2 College of Food and Agriculture, Hetao College, Bayannur 015000, China; 18686197338@163.com

**Keywords:** forage, sheep, breed, meat, metabolomic

## Abstract

This study evaluated the effects of forage source on the longissimus dorsi muscle metabolomic profiles of Dumont and Mongolian sheep. A total of 24 male sheep, including Dumont sheep (DS) and Mongolian sheep (MS), were assigned to a 2 × 2 factorial design with two breeds and two forage sources: alfalfa hay (AH) and corn straw (CS). The four groups were DSAH, DSCS, MSAH, and MSCS, with six lambs per group. Untargeted metabolomics was used to characterize muscle metabolic profiles after a 90-day feeding period. Stable metabolomic separations were mainly observed between forage treatments within each breed, namely DSAH vs. DSCS and MSAH vs. MSCS, whereas breed comparisons under the same forage condition showed limited separation and weaker model stability. OPLS-DA combined with univariate analysis identified 101 and 100 differential metabolites in the DSAH vs. DSCS and MSAH vs. MSCS comparisons, respectively. Pathway enrichment analysis indicated that forage-associated metabolic differences were mainly related to amino acid metabolism, lipid metabolism, and energy metabolism. AH feeding was associated with broader changes in muscle metabolic pathways than CS feeding, particularly in pathways related to nutrient utilization and lipid-related metabolism. These findings suggest that the forage source is closely associated with muscle metabolic variation in fattening lambs, while possible breed-related differences in metabolic responsiveness require further validation.

## Simple Summary

This study compared the longissimus dorsi muscle metabolomic profiles of Dumont and Mongolian lambs fed two different forage sources, alfalfa hay and corn straw. Clear and stable metabolic differences were mainly observed between forage treatments within each breed, whereas breed differences under the same forage condition were limited and less stable. Alfalfa hay feeding was associated with changes in metabolites and pathways mainly related to amino acid metabolism, lipid metabolism, and energy metabolism. These findings suggest that forage source may play an important role in shaping muscle metabolic profiles in fattening lambs, while possible breed-related differences in metabolic responsiveness require further validation.

## 1. Introduction

Mutton has become one of the important meat products consumed worldwide due to its high-quality protein and nutritional value. It contains all essential amino acids and is rich in minerals such as iron and zinc, as well as B vitamins [1]. In addition, lamb fat contains abundant conjugated linoleic acid (CLA) and n-3 polyunsaturated fatty acids (PUFAs), which are considered beneficial for human cardiovascular and metabolic health [1]. To meet the increasing demand for mutton consumption, the sheep industry in China has continuously promoted the development of new breeds and the upgrading of feeding technologies [2]. For example, Mongolian sheep, an ancient and high-quality meat sheep breed in northern China, are widely raised in regions such as Inner Mongolia due to their excellent meat quality and strong adaptability. Dorper sheep, originally from South Africa, are commonly used as terminal sires for high-quality hybrid meat sheep because of their rapid growth rate and high lean meat yield [3]. Dumont sheep were developed under this background as a novel meat-type sheep breed. Previous studies have shown that under identical feeding conditions, Dumont sheep and Mongolian sheep exhibit differences in growth performance and meat quality. Some nutritional indices of Dumont lambs, such as n-3 polyunsaturated fatty acids, have been reported to be lower than those of purebred Mongolian sheep, suggesting that crossbreeding improvement may have potential negative effects on meat quality [4]. However, these studies did not consider possible differences in feed utilization efficiency between the two breeds, and nutritional regulation may compensate for differences in muscle nutritional indices between them. Improving feed utilization efficiency and growth performance through optimized forage strategies is a major objective in modern sheep production, particularly for newly developed crossbred meat sheep [5].

Coarse forage constitutes the fundamental component of meat sheep diets and typically accounts for a relatively high proportion [6]. In northern China, corn is extensively cultivated, and corn straw, as its by-product, is commonly used to feed local Mongolian sheep due to its large availability and low cost. However, corn straw is characterized by high fiber content, low protein content, and poor palatability [7], which may result in long-term nutritional limitations in Dumont and Mongolian sheep during feeding. In contrast, alfalfa, as a high-quality leguminous forage, is widely used in livestock production worldwide due to its rich content of vitamins, proteins, and amino acids and plays important roles in improving animal performance, protecting liver function, and optimizing meat quality [8]. Studies replacing low-quality forage with alfalfa have reported positive effects on production performance and meat quality in various livestock species [9]. It has been reported that supplementation of alfalfa in sheep diets significantly improves fattening performance and carcass traits [10]. Regarding meat quality, alfalfa has been shown to significantly increase tenderness and the longissimus dorsi (LD) muscle area, reduce drip loss, and significantly decrease the contents of stearic acid, linoleic acid, arachidonic acid, n-6 PUFAs, and total PUFAs in muscle fatty acid composition. A study by Baldi et al. [11] demonstrated that compared with direct supplementation of vitamin E in grain-based diets, feeding alfalfa significantly improved the utilization efficiency of natural vitamin E in lambs. In studies on finishing pigs, replacing 25% of soybean meal with alfalfa leaf meal significantly improved growth performance and meat quality, enhanced antioxidant capacity, and promoted optimal physiological health, thereby improving meat yield and quality [12,13,14]. However, despite the well-documented productive and meat quality benefits of alfalfa, the underlying metabolic mechanisms through which different forage types influence muscle metabolism across sheep breeds remain insufficiently understood.

Metabolomic analysis provides a robust means of extracting biologically and agriculturally significant information from global metabolic profiles, as well as from metabolic changes triggered by diverse factors during production processes. It provides an accurate and rapid approach for screening and identifying nutritional components in meat [15,16,17]. At present, metabolomics has been widely applied to evaluate the appearance quality, eating quality, and credence quality of meat products. For instance, untargeted metabolomics using GC–MS and LC–MS/MS has been applied to identify potential biomarker metabolites in chicken serum and muscle, successfully establishing predictive models for chicken breast pH [18]. In beef cattle, metabolomics has demonstrated considerable potential for identifying predictive markers of sensory meat quality [19,20,21]. Such biomarkers are of great importance for meat phenotype evaluation [16]. This technique enables simultaneous detection of hundreds to thousands of small-molecule metabolites in biological samples. Combined with multivariate statistical analysis and metabolic pathway annotation, metabolomics can provide deep insights into how nutritional or genetic factors influence muscle metabolic networks [22]. Previous studies have applied metabolomics to investigate the effects of diets on growth performance, fattening performance, and meat quality [23]. However, most of these studies have focused on single dietary factors or single breeds, and integrative analyses combining forage type and genetic background remain scarce.

Previous studies have demonstrated that forage quality influences growth performance, meat quality, and certain metabolic traits in ruminants [9,21,23], while breed-related differences in muscle characteristics have also been widely reported [1]. However, most available studies have investigated forage effects or breed differences independently, rather than evaluating their combined influence within the same experimental framework. Comparative metabolomic evidence describing how nutritionally contrasting forages modulate muscle metabolic profiles across genetically distinct sheep breeds remains limited [22], which restricts our understanding of diet–breed interactions and their relevance to precision feeding in sheep production. From a production perspective, clarifying these metabolomic responses is important because forage-associated changes in muscle metabolism may be related to nutrient utilization efficiency, growth performance, and meat quality traits in lambs. We therefore hypothesized that replacing corn straw with alfalfa hay would alter key metabolic pathways related to amino acid, lipid, and energy metabolism in lamb muscle, and that these responses would differ between Dumont and Mongolian sheep because of differences in genetic background and forage utilization.

Accordingly, this study used a non-targeted metabolomics approach to compare the muscle metabolic profiles of Dumont and Mongolian lambs fed alfalfa hay- or corn straw-based diets, with the aim of identifying forage-associated metabolic pathways involved in nutrient utilization and muscle metabolism and providing a metabolomic basis for future studies linking these pathways with animal performance and meat quality.

## 2. Materials and Methods

### 2.1. Experimental Animals and Sample Collection

The feeding experiment was carried out from May to August 2024 at a commercial sheep farm located in Hohhot, Inner Mongolia Autonomous Region, China (40°53′ N, 111°13′ E). All experimental protocols involving animals were reviewed and approved by the Animal Welfare and Ethics Committee of Inner Mongolia Agricultural University (Approval No. NND2024076). Animal management and experimental procedures were conducted in accordance with the guidelines of the Chinese Academy of Animal Health Research (GB 14925-2010 [24]) and the European Union’s welfare guidelines (Directive 2010/63/EU [25]) on the protection of animals used for scientific purposes. The entire trial lasted 105 days, consisting of a 15-day adaptation period followed by a 90-day experimental period. All sheep were obtained from the same breeding population at Jinglai Ranch in Hohhot, Inner Mongolia, to minimize genetic and environmental variation. To avoid potential sex-related effects, only male sheep were included in the experiment. A total of 24 healthy sheep aged two months with similar initial body weight (19.90 ± 0.15 kg) were selected, including 12 Dumont sheep and 12 Mongolian sheep. Animals were randomly allocated to four treatment groups following a 2 × 2 randomized block design, namely Dumont sheep fed alfalfa hay (DSAH), Dumont sheep fed corn straw (DSCS), Mongolian sheep fed alfalfa hay (MSAH), and Mongolian sheep fed corn straw (MSCS), with six sheep assigned to each group. Two experimental diets were formulated to meet the nutrient requirements for meat sheep production as specified by the Chinese national standard (NY/T 816-2021 [26]). The diets were designed to be isoenergetic and isonitrogenous. Detailed ingredient composition and nutrient levels of the diets are provided in Supplementary Table S1. All sheep were housed individually in outdoor pens measuring 1.0 × 1.0 m^2^. Animals were offered total mixed rations (TMRs) twice daily at 09:00 and 15:00. The initial feeding allowance was set at 2% of body weight on a dry matter basis. Feeding amounts were subsequently adjusted according to daily feed refusals, maintaining residual feed levels between 5% and 10%. Throughout the experiment, animals had unrestricted access to feed and clean drinking water. To minimize potential confounding effects, all animals were obtained from the same breeding population, were of similar age and initial body weight, and were managed under the same housing and feeding conditions throughout the trial. Nevertheless, individual variation in feed intake and physiological responses could not be completely excluded under practical production conditions. At the conclusion of the feeding trial, all lambs in each group (*n* = 6 per group) were fasted for 16 h prior to slaughter and subsequently processed for sample collection. The sheep were humanely slaughtered by the farm’s professional slaughterers using the stunning and bleeding method, with stunning carried out using a humane stunning device (CASH CPK200, Accles & Shelvoke Co., Ltd., Sutton Coldfield, UK). Following slaughter, the LD muscle was excised from the left side of the carcass, flash-frozen in liquid nitrogen and used for metabolomic analysis.

### 2.2. Non-Targeted Metabolomics Analysis and Sample Preparation

#### 2.2.1. Sample Extraction Procedure

Frozen samples were removed from −80 °C storage and thawed on ice for 15–20 min prior to extraction. After thawing, 100 mg of each sample was weighed into a 2 mL centrifuge tube containing a single 6 mm stainless-steel grinding bead. Then, 800 μL of extraction solvent (methanol:water = 4:1, *v*/*v*) containing four internal standards, including L-2-chlorophenylalanine (0.02 mg/mL), was added for metabolite extraction. Samples were homogenized using a cryogenic tissue grinder (Wonbio-96c, Wonbio Co., Ltd., Shanghai, China) at −10 °C and 50 Hz for 6 min, followed by ultrasonic extraction at 5 °C and 40 kHz for 30 min in an ultrasonic bath (SBL-10DT, Scientz, Ningbo, China). The homogenates were then kept at −20 °C for 30 min to facilitate protein precipitation. Afterward, samples were centrifuged at 13,000× *g* and 4 °C for 15 min using a refrigerated centrifuge (5430 R, Eppendorf, Hamburg, Germany), and the resulting supernatants were transferred into insert-equipped sample vials for subsequent instrumental analysis. QC samples were generated by mixing equal volumes of all sample extracts and were injected after every 5–10 analytical samples to evaluate analytical repeatability and instrument stability.

#### 2.2.2. UPLC–MS Analytical Conditions

Metabolomic profiling was performed using an ultra-performance liquid chromatography system coupled with mass spectrometry (UPLC–TripleTOF 6600, SCIEX, Shanghai, China). A 3 μL aliquot of each sample was injected and separated on an HSS T3 chromatographic column (100 mm × 2.1 mm i.d., 1.8 μm particle size; Waters, Santa Barbara, CA, USA) prior to mass spectrometric detection. The mobile phase consisted of solvent A (water:acetonitrile = 95:5, *v*/*v*, containing 0.1% formic acid) and solvent B (acetonitrile:isopropanol:water = 47.5:47.5:5, *v*/*v*/*v*, containing 0.1% formic acid). Chromatographic separation was carried out at a flow rate of 0.40 mL/min, with the column temperature maintained at 40 °C. Mass spectrometric data acquisition was performed in both positive and negative ionization modes over a mass-to-charge ratio (*m*/*z*) range of 50–1200. The ion spray voltage was set to 5500 V in positive mode and −4500 V in negative mode. Additional operating parameters included a nebulizer gas pressure of 50 psi, auxiliary heater gas pressure of 13 psi, curtain gas pressure of 35 psi, and an ion source temperature of 450 °C. Collision energy was applied in a cyclic mode at 20, 40, and 60 V.

#### 2.2.3. Metabolite Data Processing

Raw LC–MS data were imported into Progenesis QI software (v3.0) (Waters Corporation, Milford, CT, USA) for integrated processing, including baseline correction, peak detection, peak integration, retention time alignment, and peak matching. This workflow produced a data matrix composed of retention time, the mass-to-charge ratio (*m*/*z*), and peak intensity. Metabolite annotation was performed by matching MS and MS/MS spectra against publicly accessible databases, including the Human Metabolome Database (HMDB) and METLIN. The resulting annotated matrix was subsequently submitted to the Majorbio Cloud Platform (cloud.majorbio.com) for further analysis. Prior to statistical analysis, the dataset was preprocessed by removing variables with excessive missing values based on the 80% rule; specifically, only variables with non-zero values detected in at least 80% of samples in any group were retained. Missing values were then replaced with the minimum value identified in the original data matrix. To minimize analytical variability caused by sample preparation and instrument performance, the dataset was normalized by total ion intensity. The formula for total ion intensity normalization is: Each value/(sum of the column/maximum sum of the column) (Note: The relative content value obtained by dividing each chromatographic peak by a coefficient, where the coefficient is the sum of the signal intensities of all peaks in each sample divided by the maximum sum of the signal intensities across all samples). Additionally, variables with a relative standard deviation (RSD) greater than 30% in the quality control samples were excluded. Finally, the normalized data were subjected to a logarithmic (log10) transformation to generate a processed dataset for subsequent analysis.

### 2.3. Statistical Analysis

Following data preprocessing, four planned pairwise metabolomic comparisons were defined according to the 2 × 2 experimental design. The DSAH vs. DSCS and MSAH vs. MSCS comparisons were used to evaluate the effects of forage source within each breed, whereas the DSAH vs. MSAH and DSCS vs. MSCS comparisons were used to explore breed-related differences under the same forage condition. Multivariate statistical analyses were performed in the R environment using the ropls package (Version 1.6.2). Principal component analysis (PCA) was first used to evaluate the overall distribution and clustering patterns of the samples. Orthogonal partial least squares discriminant analysis (OPLS-DA) was then performed for the planned pairwise comparisons to further assess group separation and to identify metabolites contributing to treatment discrimination. To evaluate model robustness and reduce the risk of overfitting, OPLS-DA models were assessed using seven-fold cross-validation. Model performance was further evaluated using R^2^Y, Q^2^, and permutation testing, and the corresponding validation results are provided in Supplementary Table S2 and Figure S2. Based on the PCA patterns and OPLS-DA validation results, only comparisons showing stable group separation and acceptable model performance were retained for differential metabolite screening and pathway enrichment analysis. Comparisons showing limited separation and weak predictive ability were considered exploratory and were not used for conclusive differential metabolite interpretation. Differential metabolites were screened using the combined criteria of VIP > 1 derived from the OPLS-DA model, fold change (FC) ≥ 1.10 or ≤ 0.91, and Student’s *t*-test *p* < 0.05. To improve transparency regarding multiple testing, *p*-values were adjusted using the Benjamini–Hochberg method during data analysis, and the adjusted *p*-values were reported in the supplementary metabolite table. Because the sample size in each group was relatively small (*n* = 6), the metabolomic results were interpreted with appropriate caution. Functional annotation of differential metabolites was conducted using the KEGG database (https://www.kegg.jp/kegg/pathway.html, accessed on 21 September 2025) to identify associated metabolic pathways. Pathway enrichment analysis was performed using the scipy.stats package in Python (Version 1.6.2), and Fisher’s exact test was applied to determine significantly enriched biological pathways related to the retained comparisons.

Growth performance data were analyzed using the PROC GLM procedure in SAS 9.4 (SAS Institute Inc., Cary, NC, USA) under a 2 × 2 factorial design. The model included breed, forage, and their interaction (breed × forage) as fixed effects. When a significant interaction was detected, Tukey’s Honest Significant Difference (HSD) test was used for multiple comparisons to distinguish between means. Results are presented as means with their corresponding standard errors of the mean (SEM). *p* ≤ 0.05 was considered statistically significant, while 0.05 ≤ *p* < 0.10 was considered a trend. Detailed growth performance data are provided in Supplementary Table S4.

## 3. Results

### 3.1. QC Sample Analysis

**Figure 1:**
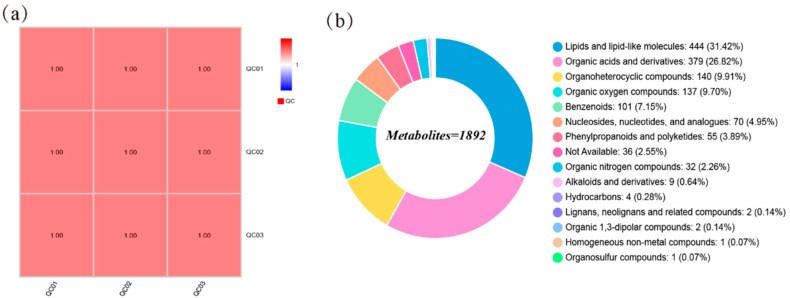
Analysis of Muscle Metabolomic Characteristics in Dumont and Mongolian Sheep in Response to Different Forage Diets. (**a**) Correlation of QC samples; (**b**) Classification of metabolites.

As shown in Figure 1a, the Pearson correlation coefficients of all quality control (QC) samples satisfied R^2^ > 0.99. In addition, QC samples clustered closely in the PCA space (Figure S1), indicating excellent analytical reproducibility and instrumental stability throughout the analytical sequence. These QC results demonstrated high analytical repeatability and low instrumental drift throughout the analytical sequence, confirming that the metabolomic dataset was reliable and suitable for subsequent multivariate and differential metabolite analyses.

### 3.2. Detection, Identification, and PCA of Metabolites

A total of 4434 and 5793 metabolic feature peaks were detected in the positive ion mode and negative ion mode, respectively, in LD muscle samples. Following database annotation, 1892 metabolites were identified. These metabolites were classified into the following categories: lipids and lipid-like molecules (444, 31.42%), organic acids and derivatives (379, 26.82%), organoheterocyclic compounds (140, 9.91%), organic oxygen compounds (137, 9.70%), benzenoids (101, 7.15%), nucleosides, nucleotides, and analogues (70, 4.95%), phenylpropanoids and polyketides (55, 3.89%), organic nitrogen compounds (32, 2.26%), alkaloids and derivatives (9, 0.64%), hydrocarbons (4, 0.28%), organic 1,3-dipolar compounds (2, 0.14%), lignans, neolignans, and related compounds (2, 0.14%), organosulfur compounds (1, 0.07%), homogeneous non-metal compounds (1, 0.07%), and other metabolites (36, 2.55%) (Figure 1b).

**Figure 2:**
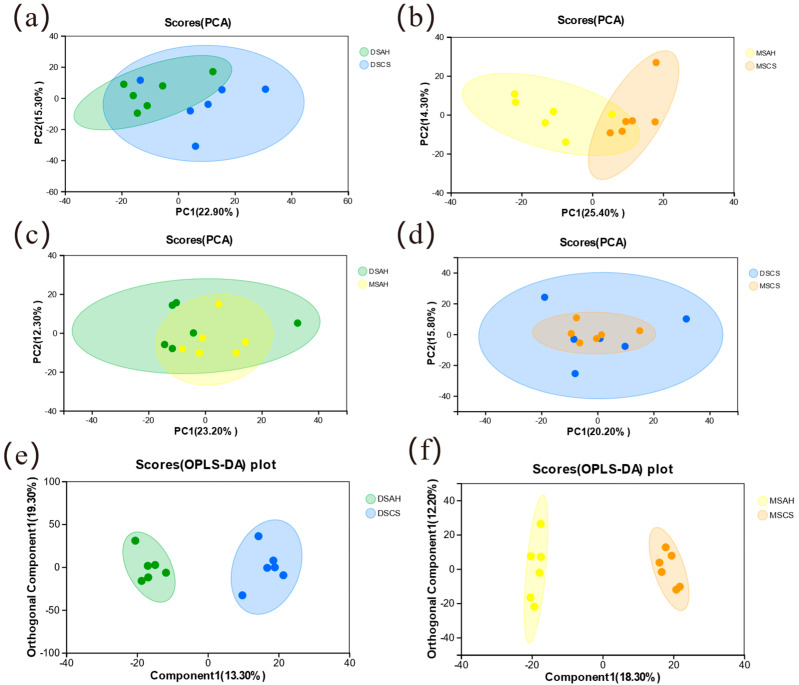
PCA and OPLS-DA of Muscle Metabolomics Data from Dumont and Mongolian Sheep in Response to Different Forages. Four-group PCA (**a**) DSAH vs. DSCS; (**b**) MSAH vs. MSCS; (**c**) DSAH vs. MSAH; (**d**) DSCS vs. MSCS. OPLS-DA analysis of two comparison groups (**e**) DSAH vs. DSCS; (**f**) MSAH vs. MSCS.

Prior to the differential metabolite analysis, principal component analysis (PCA) was performed to evaluate the variation among samples, and the model quality assessment parameters are presented in Table S2. As shown in Figure 2a–d, LD muscle samples from the four treatment groups exhibited similar metabolomic characteristics within each group, whereas differences were observed among groups along the principal components. Specifically, the DSAH vs. DSCS and MSAH vs. MSCS groups showed significant separation along the first principal component (PC1) and second principal component (PC2) (*p* < 0.05), indicating that forage type exerted a strong influence on the muscle metabolomic profile. In contrast, considerable overlap was observed between the DSAH vs. MSAH and DSCS vs. MSCS groups under the same dietary conditions. This overlap suggests that breed background had a weaker effect than forage type on the overall metabolic pattern of LD muscle under the present experimental conditions. To further identify differential metabolites, OPLS-DA was performed on the identified metabolites in muscle tissues. The OPLS-DA models were further evaluated by permutation testing. The results indicated acceptable model fit and predictive performance for the DSAH vs. DSCS and MSAH vs. MSCS comparisons, whereas the DSAH vs. MSAH and DSCS vs. MSCS models showed limited predictive ability and a higher risk of overfitting (Table S2, Figure 2e,f). Subsequent analyses were conducted using data from both positive and negative ion modes. The results demonstrated that the DSAH vs. DSCS and MSAH vs. MSCS comparison groups exhibited clear separation, and the models showed good data fitting, strong predictive ability, and a low risk of overfitting (Table S2, Figure S2). However, the DSAH vs. MSAH and DSCS vs. MSCS comparison groups exhibited poor predictive ability and a relatively high risk of overfitting. Combined with the absence of clear separation observed in PCA, these findings indicate that no statistically significant and stable metabolic differences were present between the DSAH vs. MSAH and DSCS vs. MSCS groups. Therefore, subsequent analyses were conducted only on the DSAH vs. DSCS and MSAH vs. MSCS comparison groups.

### 3.3. Screening and Analysis of Differential Metabolites

**Figure 3:**
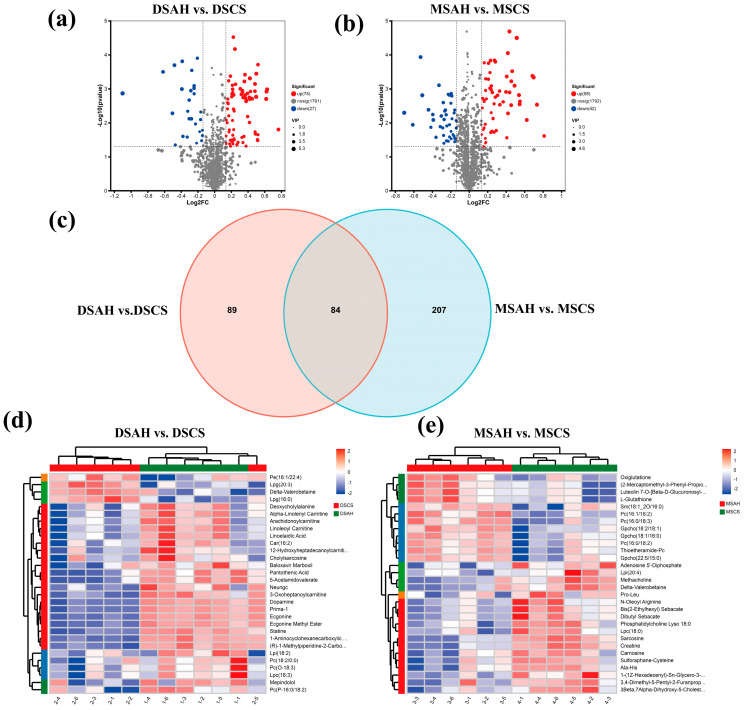
Analysis of differential metabolites in the muscle tissue of Dumont and Mongolian sheep under different forage diets. (**a**,**b**) show the volcano plots for DSAH vs. DSCS group and MSAH vs. MSCS group. (**c**) shows the Venn diagram of differential metabolites. (**d**,**e**) show the heatmaps of differential metabolites for DSAH vs. DSCS group and MSAH vs. MSCS group.

Pairwise comparisons of metabolites were conducted among the four treatment groups. Differential metabolites were screened using the predefined exploratory criteria of VIP > 1, FC ≥ 1.10 or ≤ 0.91, and Student’s *t*-test *p* < 0.05, with Benjamini–Hochberg-adjusted *p*-values reported in the supplementary metabolite table for transparency. The volcano plot analysis revealed that a total of 101 differential metabolites were identified in the DSAH vs. DSCS comparison (74 upregulated and 27 downregulated), while 100 differential metabolites were identified in the MSAH vs. MSCS comparison (58 upregulated and 42 downregulated) (Figure 3a,b). Among the 101 differential metabolites identified in the DSAH vs. DSCS comparison, the metabolites included 17 lipids and lipid-like molecules, 19 amino acids and their derivatives, 14 phenylpropanoids and flavonoids, 8 organic acids and their derivatives, 7 nucleotides and their derivatives, 6 alkaloids and their derivatives, 5 glycosides and carbohydrate derivatives, and 13 heterocyclic and unclassified compounds. In the MSAH vs. MSCS comparison, a total of 100 differential metabolites were identified, including 32 lipids and lipid-like molecules, 37 amino acids and their derivatives, 9 phenylpropanoids and flavonoid compounds, 12 organic acids and their derivatives, 8 alkaloids and their derivatives, 6 glycosides and carbohydrate derivatives, and 17 heterocyclic and unclassified compounds. Further analysis using a Venn diagram demonstrated that 84 differential metabolites were shared between the two comparison groups (DSAH vs. DSCS and MSAH vs. MSCS) (Figure 3c). The large number of shared differential metabolites suggests that the metabolic response to forage type was largely conserved across breeds. Functionally, these shared metabolite changes were mainly associated with amino acid, lipid, and energy metabolism, further supporting the view that forage source was the dominant driver of metabolic reprogramming in LD muscle. Hierarchical clustering analysis of differential metabolites revealed significant differences in metabolite abundance patterns among the four groups (Figure 3d,e).

### 3.4. Metabolic Pathway Enrichment Analysis of Differential Metabolites

**Figure 4:**
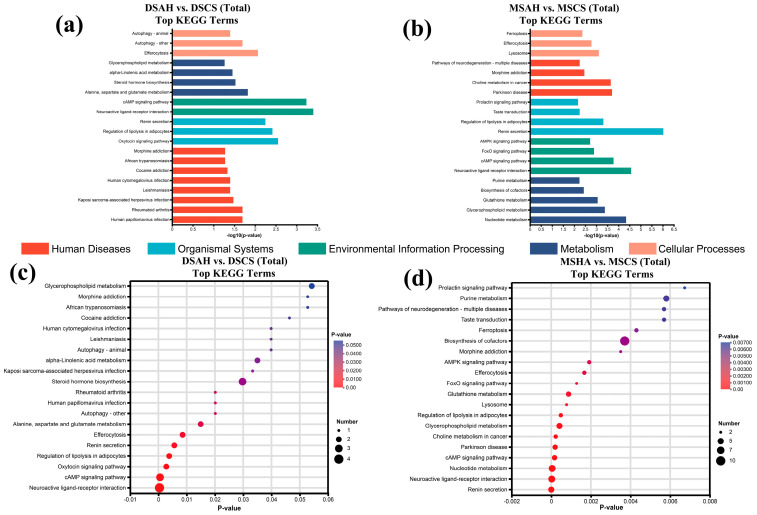
Metabolic pathway maps of muscle metabolites in Dumont and Mongolian sheep under different forage feeding conditions. (**a**,**b**) show the categories of the top 20 KEGG pathways identified by enrichment analysis in the DSAH and DSCS groups, and the MSAH and MSCS groups, respectively; (**c**,**d**) show the top 20 KEGG pathways identified by enrichment analysis in the DSAH and DSCS groups, and the MSAH and MSCS groups, respectively.

Figure 4 further illustrates the specific metabolic pathways through which AH and CS influenced muscle metabolism in the two lamb breeds. In the DSAH vs. DSCS comparison, differential metabolites were significantly enriched in 13 metabolic pathways, mainly including the cAMP signaling pathway; regulation of lipolysis in adipocytes; alanine, aspartate and glutamate metabolism; steroid hormone biosynthesis; and α-linolenic acid metabolism. In the MSAH vs. MSCS comparison, differential metabolites were annotated to a total of 32 metabolic pathways, primarily including glycerophospholipid metabolism, regulation of lipolysis in adipocytes, arachidonic acid metabolism, arginine biosynthesis, and alanine, aspartate and glutamate metabolism pathways. Notably, the significantly enriched pathways were mainly related to amino acid, lipid, and energy metabolism, suggesting that the metabolomic differences between forage treatments were primarily associated with nutrient utilization in muscle tissue. These metabolic differences may help to explain the growth performance patterns observed in Supplementary Table S4, although direct metabolite–phenotype associations were not evaluated in the present study.

## 4. Discussion

Untargeted metabolomics analysis enables comprehensive characterization of metabolites in muscle tissue and provides insight into muscle metabolic status. In the present study, PCA and OPLS-DA consistently indicated that forage type exerted a stronger influence on the muscle metabolome than breed background. Moreover, the major forage-associated differences were concentrated in pathways related to amino acid metabolism, lipid metabolism, and energy metabolism. These observations represent the main data-supported findings of the present study and indicate that forage quality was a major driver of metabolic variation in LD muscle.

Within the Neuroactive ligand–receptor interaction and cAMP signaling pathway, dopamine, epinephrine, and prostaglandin E2 (PGE2) were significantly upregulated in muscle tissues of both lamb breeds under AH feeding conditions. Similarly, epinephrine and PGE2 were also enriched in the regulation of lipolysis in the adipocyte pathway. Because these metabolites were involved in multiple pathways and consistently increased under AH feeding, they may represent important components of the forage-associated metabolic response in LD muscle. PGE2 is a bioactive lipid mediator involved in inflammation, angiogenesis, and tissue regulation [27,28]. Previous studies have shown that PGE2 can participate in cAMP-related signaling and lipid metabolic regulation [29,30,31]. Therefore, the higher abundance of PGE2 in the AH groups may suggest altered lipid signaling and metabolic regulation in muscle tissue. The higher abundance of PGE2 in AH-fed lambs may be related to differences in dietary nutrient composition or bioactive compounds; however, this hypothesis requires targeted validation of prostaglandin metabolism and related regulatory enzymes [32,33]. However, the present study did not directly examine the underlying regulatory mechanisms or the direct effects of PGE2 on muscle growth.

Similarly, epinephrine is known to participate in pathways related to energy mobilization and metabolic regulation [34,35]. Its increased abundance in the AH groups may therefore reflect differences in nutrient-responsive metabolic activity between forage treatments. These changes may reflect forage-associated differences in nutrient-responsive energy metabolism, but the present untargeted metabolomics data do not allow direct confirmation of endocrine or adrenergic regulatory mechanisms [36].

In the alanine, aspartate and glutamate metabolism pathway, both comparison groups showed similar changes. Among the differential metabolites, N6-(1,2-dicarboxyethyl) AMP was significantly upregulated in both comparisons, whereas N-acetylaspartylglutamic acid (NAAG) increased only in the DSAH vs. DSCS comparison. Because these metabolites are linked to amino acid and nucleotide metabolism, their changes may reflect forage-associated differences in muscle nutrient metabolism [37,38].

An important finding of the present study is that metabolic differences between breeds under the same forage condition were limited, whereas the responses to forage type within each breed were more apparent. This pattern suggests that forage source exerted a stronger influence on muscle metabolism than breed background under the present experimental conditions. At the same time, Mongolian lambs showed a broader metabolic response to AH feeding than Dumont lambs, particularly in pathways related to energy metabolism, antioxidant metabolism, and glycerophospholipid metabolism. One possible explanation is that Mongolian sheep, as a native breed historically adapted to lower-quality forage resources, may be more metabolically responsive to improved dietary quality, whereas Dumont sheep may exhibit relatively greater metabolic stability. However, these interpretations remain tentative and should be validated in larger studies.

Although no statistically significant metabolic differences were observed between breeds under identical feeding conditions (DSAH vs. MSAH and DSCS vs. MSCS), metabolic differences were detected between forage treatments within each breed (DSAH vs. DSCS and MSAH vs. MSCS). This pattern suggests that the metabolic response to forage type may vary between breeds, even though breed-related differences under the same dietary condition were limited. In particular, Mongolian lambs showed a broader metabolic response to AH feeding than Dumont lambs, with more pronounced changes in pathways related to energy metabolism, lipid metabolism, antioxidant metabolism, and amino acid metabolism. For example, the MSAH group showed increased levels of ATP, AMP, and ADP, as well as higher abundances of oxidized glutathione (GSSG), reduced glutathione (GSH), cysteinylglycine, γ-glutamylcysteine, and several glycerophospholipid-related metabolites. These findings may indicate a stronger metabolic adjustment to AH feeding in Mongolian lambs.

One possible explanation is that differences in genetic background and long-term adaptation to available forage resources may have contributed to the distinct metabolic responses of the two breeds. AH is a high-quality forage rich in protein and bioactive compounds, whereas CS has relatively lower nutritional value and higher fiber content [39,40,41,42,43,44,45,46]. As a native breed in northern China, Mongolian sheep may be more responsive to improved dietary quality, while Dumont sheep may show relatively more stable metabolic responses under the same experimental conditions [47,48]. However, these interpretations remain tentative. Considering the limited sample size and the absence of direct mechanistic validation, further studies are needed to confirm whether the observed breed-related differences in responsiveness to forage are stable and biologically meaningful.

Although the analyses of DSAH vs. DSCS and MSAH vs. MSCS comparisons revealed metabolic differences between Dumont and Mongolian lambs under different forage conditions, forage type remained the primary factor influencing muscle metabolic variation in both breeds. Overall, the results indicate that forage nutritional quality plays a central role in shaping muscle metabolic responses, with high-quality forage supporting more extensive metabolic adjustments. Specifically, AH was associated with changes in pathways related to amino acid metabolism, lipid metabolism, and energy metabolism. These metabolomic patterns were broadly consistent with the growth performance patterns shown in Supplementary Table S4, suggesting that forage quality may influence nutrient utilization and growth-related responses in growing lambs. However, these interpretations should be made cautiously because direct associations between specific metabolites and productive traits were not examined in the present study. Future studies should explore mixed feeding strategies using different proportions of alfalfa and CS to further evaluate their potential effects on growth performance, meat quality, and feeding cost.

## 5. Conclusions

In summary, the present untargeted metabolomics analysis suggests that the forage source was closely associated with LD muscle metabolic profiles in Dumont and Mongolian lambs. Stable metabolic differences were mainly observed in the within-breed forage comparisons, namely DSAH vs. DSCS and MSAH vs. MSCS, whereas breed comparisons under the same forage condition showed limited separation and were not used for conclusive interpretation. The main forage-associated pathways were related to amino acid metabolism, lipid metabolism, and energy metabolism. Exploratory comparison of the two within-breed forage responses suggested that Mongolian lambs may exhibit broader metabolic adjustments to AH feeding than Dumont lambs; however, this breed-related pattern requires further validation. These findings provide metabolomic evidence that forage quality may influence muscle metabolic variation in fattening lambs. Further studies with larger sample sizes and integrated analyses of metabolomic profiles, growth performance, and meat quality are needed to validate these observations and clarify their practical implications for sheep production.

## Data Availability

The original contributions presented in this study are included in the article/Supplementary Material. Further inquiries can be directed to the corresponding authors.

## Supplementary Materials

The following supporting information can be downloaded at: https://www.mdpi.com/article/10.3390/ani16101487/s1, Table S1. Ingredient composition and nutritional value of experimental feed (dry matter basis). Table S2. Metrics for evaluating the quality of PCA and OPLS-DA models. Table S3. The Effects of Different Roughage Types on Metabolic Differences in the Muscle Metabolome of Dumont and Mongolian Sheep. Table S4. Effects of Roughage Sources on Growth Performance in Dumont Sheep and Mongolian Sheep. Figure S1. PAC Analysis of QC Samples. Figure S2. Plot of OPLS-DA permutation of the DSAH vs. DSCS; MSAH vs. MSCS; DSAH vs. MSAH; DSCS vs. MSCS.
