# Supplementary material for: Muscle Metabolomic Responses of Dumont and Mongolian Sheep to Alfalfa Hay- and Corn Straw-Based Diets: An Untargeted Metabolomics Study
Source: Animals (Basel). 2026 May 12;16(10):1487. doi: 10.3390/ani16101487 (PMC13203844; doi:10.3390/ani16101487)
Supplement: Supplementary file 1 [file animals-16-01487-s001.zip › animals-4264543-supplementary.pdf]

Supplementary Material

Table S1

Ingredient composition and nutritional value of experimental feed (dry matter basis).

Ingredients	Treatments[1]		Nutrient	Treatments	
	AH	CS		AH	CS
Corn stalk	0	50	Dry matter	87.46	87.4
Alfalfa hay	50	0	Digestible energy, MJ/kg	12.03	12.05
Corn	28.2	26.5	Crude protein	13.23	13.13
Rapeseed meal	0	15.1	Starch	20.35	20.47
Soybean meal	0	0	Acid detergent fiber	20.79	27.82
Corn husk	12.5	0	Neutral detergent fiber	35.61	45.13
Wheat bran	6	0	Ether extract	3.68	3.67
Soybean oil	0.3	0.4	Feed cost, USD/t	450	290
Salt	0.5	0.5	Rumen degradable protein	6.4	5.2
Premix[2]	2.5	2.5			

[1] AH: alfalfa hay diet, CS: corn stalk diet. [2] The premix contained/kg diet: vitamin A 6000 IU, vitamin D3 2000 IU, vitamin E 15 IU, vitamin K3 1.8 mg, vitamin B1 0.35 mg, vitamin B2 8.5 mg, vitamin B6 0.9 mg, vitamin B12 0.03 mg, D-pantothenic acid 16 mg, nicotinic acid 22 mg, folic acid 1.5 mg, biotin 0.15 mg, Cu 8 mg, Fe 40 mg, Mn 20 mg, Zn 40 mg, I 0.8 mg, Se 0.3 mg, and Co 0.3 mg.

Table S2

Metrics for evaluating the quality of PCA and OPLS-DA models

Item	R^2X(cum)	R^2Y(cum)	Q^2(cum)	Title[1]
PCA	0.512	-	-	DSAH vs DSCS
	0.522	-	-	MSAH vs MSCS
	0.469	-	-	DSAH vs MSAH
	0.48	-	-	DSCS vs MSCS
OPLS-DA	0.422	0.999	0.331	DSAH vs DSCS
	0.29	0.99	0.557	MSAH vs MSCS
	0.303	0.979	0.12	DSAH vs MSAH
	0.257	0.992	0.0332	DSCS vs MSCS

1 AH, alfalfa silage; CS, corn straw; Dumont sheep, DS; Mongolian sheep, MS.

Table S3

The Effects of Different Roughage Types on Metabolic Differences in the Muscle Metabolome of Dumont and Mongolian Sheep

Metabolite	Formula	VIP	Log2FC	*p*-value	Regulate
**DSAH vs DSCS**					
3-Caffeoyl-1,5-Quinolactone	C16H16O8	2.6203	0.5218	0.000195	up
Tranexamic Acid	C8H15NO2	2.6046	0.2262	3.02E-05	up
4-[2-[[2-(4-Hydroxyphenyl)Acetyl]Amino]Ethylamino]-4-Oxobut-2-Enoic Acid	C14H16N2O5	2.5957	-0.3856	0.000156	down
Hypoglycin B	C12H18N2O5	2.5838	-0.2049	0.000127	down
Statine	C8H17NO3	2.5649	0.2452	6.82E-05	up
Supinidine	C8H13NO	2.5329	0.4856	0.000724	up
N-(6-Cyano-3-Hydroxy-2,2-Dimethyl-3,4-Dihydrochromen-4-Yl)-N-Methylethanesulfonamide	C15H20N2O4S	2.5034	-0.6158	0.000319	down
2-(4-Acetyl-6,8-Dihydroxy-1-Oxoisoquinolin-2-Yl)-4-Methylpentanoic Acid	C17H19NO6	2.4882	0.4847	0.001177	up
N-Methyl-2,3-Dihydro-1H-Inden-2-Amine	C10H13N	2.4778	-0.4792	0.000202	down
6-Hydroxypseudooxynicotine	C10H14N2O2	2.4634	-0.2435	0.000819	down
Trans-Zeatin	C10H13N5O	2.4602	0.3665	0.001533	up
Lycopersiconol	C21H34O3	2.4588	0.5014	0.000361	up
1-(2-Methoxyphenyl)Ethanone	C9H10O2	2.4566	-0.2716	0.000286	down
(-)-Epiafzelechin 3-Gallate	C22H18O9	2.4551	0.4319	0.001197	up
Dhv-Pge2	C22H34O5	2.4367	0.3656	0.000386	up
1-Aminocyclohexanecarboxylic Acid	C7H13NO2	2.4231	0.1914	0.000428	up
Maresin 1	C22H32O4	2.4194	0.4247	0.000496	up
21-Hydroxy-5B-Pregnane-3,11,20-Trione	C21H30O4	2.3918	0.3584	0.000613	up
(R)-1-Methylpiperidine-2-Carboxylic Acid	C7H13NO2	2.3869	0.1703	0.000587	up
4-Amino-3-Hydroxybutanoylcarnitine	C11H22N2O5	2.3866	-1.1005	0.001363	down
1-(4-Hydroxypentyl)Indole-3-Carboxylic Acid	C14H17NO3	2.3812	0.2154	0.000792	up
Epigallocatechin	C15H14O7	2.3807	0.1562	0.003936	up
Lysinoalanine	C9H19N3O4	2.3682	0.2528	0.00074	up
8-Nonenoylglycine	C11H19NO3	2.3497	0.3099	0.001005	up
Ecgonine Methyl Ester	C10H17NO3	2.3491	0.2177	0.001034	up
Pro-Lys	C11H21N3O3	2.3481	-0.3902	0.001021	down
Phenylalanyl-Alanine	C12H16N2O3	2.3439	-0.2441	0.000963	down
8-Acetamido-2-Methyl-7-Oxononanoic Acid	C12H21NO4	2.3431	0.6307	0.00105	up
N-(1,3-Dihydroxy-3-Methylpentan-2-Yl)-2-Methylhexa-2,4-Dienamide	C13H23NO3	2.3386	0.3356	0.001144	up
Adp Ribose	C15H23N5O14P2	2.3379	0.3704	0.001868	up

4-Methoxymelilotic Acid	C10H12O4	2.3321	-0.1643	0.004844	down
1,2,4-Trideoxy-3-C-Methyl-4-{[(2E,4E)-2-Methyl-2,4-Hexadienoyl]Amino}Pentitol	C13H23NO3	2.33	0.6239	0.001204	up
Alpha-Methyltryptamine	C11H14N2	2.3262	-0.2822	0.001136	down
Sabine	C27H45NO7	2.3218	-0.5034	0.005294	down
Ecgonine	C9H15NO3	2.3215	0.2188	0.001301	up
Dattelic Acid	C16H16O8	2.3201	0.2362	0.004531	up
3-Hydroxy-N-(2-Oxooxolan-3-Yl)Hexanamide	C10H17NO4	2.3187	0.3358	0.001241	up
1-(3-Hydroxy-7-Azaspiro[3.5]Nonan-7-Yl)-2-Methoxyethanone	C11H19NO3	2.3107	0.3222	0.001454	up
Prima-1	C9H15NO3	2.3099	0.2096	0.001433	up
(+/-)-Octanoylcarnitine	C15H29NO4	2.3077	0.3954	0.001368	up
Dopamine	C8H11NO2	2.3056	0.2306	0.00152	up
Sterebin E	C20H34O4	2.2975	0.4999	0.001721	up
Adenosine Diphosphate Ribose	C15H23N5O14P2	2.297	0.2074	0.001715	up
5-Hydroxymebendazole	C16H15N3O3	2.2966	-0.2433	0.001402	down
5-Methoxy Methylone	C12H15NO4	2.2926	0.4188	0.001794	up
Celgosivir	C12H21NO5	2.2913	0.3434	0.001801	up
1-(3-Hydroxy-7-Azaspiro[3.5]Nonan-7-Yl)-3-Methoxypropan-1-One	C12H21NO3	2.2836	0.6201	0.001988	up
3-Hydroxysebacic Acid	C10H18O5	2.272	0.4255	0.002002	up
Neuroprotectin D1	C22H32O4	2.2716	0.3824	0.002209	up
2-N-Propyl-4-Oxopentanoic Acid	C8H14O3	2.2687	0.4501	0.001954	up
Coumaroyl Quinic Acid	C16H18O8	2.2663	0.3976	0.006607	up
N-(4-Oxopentyl)Acetamide	C7H13NO2	2.2569	0.1608	0.002	up
L-Pyridosine	C12H18N2O4	2.2493	-0.303	0.002149	down
Cis-4-Hydroxycyclohexylacetic Acid	C8H14O3	2.2328	0.3784	0.002368	up
Proline Betaine	C7H13NO2	2.2325	0.1578	0.002579	up
Corilagin	C27H22O18	2.2307	0.1427	0.008307	up
3-(Imidazo[4,5-B]Pyridin-3-Ylmethyl)-N-Propan-2-Ylpiperidine-1-Carboxamide	C16H23N5O	2.2204	0.1651	0.002763	up
Testosterone Glucuronide	C25H36O8	2.2178	-0.3021	0.007638	down
N1-(5-Phospho-D-Ribosyl)-Amp	C15H23N5O14P2	2.2001	0.2284	0.003992	up
Procyanidin B2	C30H26O12	2.1575	0.4198	0.009789	up
3-Oxoheptanoylcarnitine	C14H25NO5	2.1018	0.1714	0.006293	up
Glutamylleucylarginine	C17H32N6O6	2.0952	-0.3198	0.004609	down
Rhodamine 6G	C28H30N2O3	2.0623	-0.2754	0.007672	down
Methyl Gallate	C8H8O5	2.0586	0.4865	0.01852	up
Indolophenanthridine	C19H18N2	2.0546	-0.1664	0.00809	down
2-Phospho-4-(Cytidine 5'-Diphospho)-2-C-Methyl-D-Erythritol	C14H26N3O17P3	2.0498	-0.1433	0.01664	down
5-Amino-3-(4-Methoxyphenyl)-5-Oxopentanoic Acid	C12H15NO4	2.0456	0.1789	0.01611	up

Cyclic Adp-Ribose	C15H21N5O13P2	2.0359	0.2165	0.008532	up
4-Caffeoylquinic Acid	C16H18O9	2.0289	0.353	0.02542	up
Gallic Acid	C7H6O5	2.0157	0.5212	0.02264	up
Deoxycholylproline	C29H47NO5	2.0102	0.1532	0.009957	up
Dihydronicotinamide Formycin Dinucleotide	C21H29N7O14P2	1.9863	0.2144	0.03625	up
Glycoursodeoxycholic Acid	C26H43NO5	1.9819	-0.2268	0.01084	down
Pro-Tyr-Ser	C17H23N3O6	1.9774	0.2303	0.01589	up
Isoquercetin	C21H20O12	1.9679	0.7663	0.01577	up
(S)-A-Amino-2,5-Dihydro-5-Oxo-4-Isoxazolepropanoic Acid N2-Glucoside	C12H18N2O9	1.9596	0.3279	0.02721	up
P-Coumaric Acid Glucuronide	C15H16O9	1.9325	0.3778	0.04899	up
Valtrate	C10H12O5	1.9285	-0.1774	0.01843	down
Altenusin	C15H14O6	1.9084	0.2359	0.02928	up
6-Octenoylcarnitine	C15H27NO4	1.8994	0.174	0.01883	up
Humilixanthin	C14H18N2O7	1.8923	0.4898	0.02141	up
(-)-Pinoresinol	C26H32O11	1.8811	0.2126	0.03182	up
Salicyluric Acid	C9H9NO4	1.8537	0.5156	0.03157	up
Vitamin P	C27H30O16	1.8529	0.3414	0.03858	up
(9S,10E,12S,13S)-9,12,13-Trihydroxyoctadec-10-Enoylcarnitine	C25H47NO7	1.8344	0.2197	0.02339	up
Sl(12:2_O/13:1_O)	C25H45NO6S	1.8318	-0.144	0.02406	down
3A,6B,7A,12A-Tetrahydroxy-5B-Cholanoic Acid	C24H40O6	1.8214	0.1398	0.0247	up
Pro-Val	C10H18N2O3	1.8114	-0.3775	0.02532	down
12-Oxo-Pda	C18H28O3	1.7494	-0.1546	0.03383	down
Urolithin B 3-O-Glucuronide	C19H16O9	1.7397	0.2817	0.03804	up
Met-Ile	C11H22N2O3S	1.7382	0.5185	0.03337	up
(2S)-2-Hydroxy-2-(Propan-2-Yl)Butanedioylcarnitine	C14H25NO7	1.7357	0.2387	0.03207	up
N6-(1,2-Dicarboxyethyl)-Amp	C14H18N5O11P	1.7322	0.1888	0.03298	up
Oleoyl-L-Carnitine	C25H47NO4	1.7129	0.1837	0.04204	up
Lepidimoic Acid	C12H18O10	1.712	0.2228	0.04211	up
Ser-Asn-Tyr	C16H22N4O7	1.7042	0.2698	0.04473	up
Isosakuranetin	C16H14O5	1.6887	-0.3261	0.02653	down
S-(1,2-Dicarboxyethyl)Glutathione	C14H21N3O10S	1.6868	0.1414	0.04153	up
Adamantylamide-Alanyl-Isoglutamine	C18H30N4O3	1.6706	-0.2137	0.03996	down
4-[(2,4-Dihydroxy-3,3-Dimethylbutanoyl)Amino]Butanoic Acid	C10H19NO5	1.6362	0.1933	0.04941	up
Homoeriodictyol	C16H14O6	1.5329	-0.4689	0.04526	down
**MSAH vs MSCS**					
Adenosine Triphosphate	C10H16N5O13P3	2.3202	0.1643	0.000171	up

2-(4-Acetyl-6,8-Dihydroxy-1-Oxoisoquinolin-2-Yl)-4-Methylpentanoic Acid	C17H19NO6	2.2406	0.6875	0.000424	up
Deoxyguanosine Triphosphate	C10H16N5O13P3	2.2366	0.2097	0.000525	up
6-Hydroxypseudooxynicotine	C10H14N2O2	2.1246	-0.1877	0.001617	down
2'-Deoxyguanosine-5'-Triphosphate	C10H16N5O13P3	2.1242	0.284	0.00176	up
21-Hydroxy-5B-Pregnane-3,11,20-Trione	C21H30O4	2.0683	0.4376	2.06E-05	up
Hypoglycin B	C12H18N2O5	2.0559	-0.1559	0.004238	down
Maresin 1	C22H32O4	2.0483	0.5163	3.18E-05	up
Supinidine	C8H13NO	2.0178	0.4561	0.003056	up
5-Hydroxymebendazole	C16H15N3O3	2.0009	-0.2632	0.004539	down
Lysinoalanine	C9H19N3O4	1.9991	0.42	8.98E-05	up
Succinylcholine	C14H30N2O4+2	1.993	-0.5259	0.000117	down
Arenobufagin	C24H32O6	1.9862	-0.2568	0.004269	down
Salicyluric Acid	C9H9NO4	1.9821	0.7355	0.002962	up
3-Oxoheptanoylcarnitine	C14H25NO5	1.982	0.2847	0.00016	up
1-Aminocyclohexanecarboxylic Acid	C7H13NO2	1.9819	0.2325	0.000147	up
(R)-1-Methylpiperidine-2-Carboxylic Acid	C7H13NO2	1.9691	0.2107	0.000176	up
Statine	C8H17NO3	1.9673	0.2767	0.000147	up
4-[2-[[2-(4-Hydroxyphenyl)Acetyl]Amino]Ethylamino]-4-Oxobut-2-Enoic Acid	C14H16N2O5	1.9653	-0.2481	0.005858	down
Testosterone Glucuronide	C25H36O8	1.947	-0.3011	0.006362	down
N-(6-Cyano-3-Hydroxy-2,2-Dimethyl-3,4-Dihydrochromen-4-Yl)-N-Methylethanesulfonamide	C15H20N2O4S	1.94	-0.3931	0.00576	down
Pindolol	C14H20N2O2	1.9372	-0.7032	0.005116	down
Lycopersiconol	C21H34O3	1.9293	0.4668	0.000305	up
1-(4-Hydroxypentyl)Indole-3-Carboxylic Acid	C14H17NO3	1.925	0.2263	0.000336	up
4-Caffeoylquinic Acid	C16H18O9	1.9217	0.2396	0.005091	up
8-Acetamido-2-Methyl-7-Oxononanoic Acid	C12H21NO4	1.9152	0.6994	0.000466	up
Celgosivir	C12H21NO5	1.9143	0.4076	0.000439	up
Dihydronicotinamide Formycin Dinucleotide	C21H29N7O14P2	1.9133	0.1751	0.01103	up
Cyclic Apt	C10H12N5O5PS	1.9101	0.2599	0.007529	up
3-(Imidazo[4,5-B]Pyridin-3-Ylmethyl)-N-Propan-2-Ylpiperidine-1-Carboxamide	C16H23N5O	1.9079	0.1797	0.000513	up
Mespirenone	C25H30O4S	1.8931	-0.3878	0.008264	down
Cinnamoylglycine	C11H11NO3	1.8923	-0.141	0.008594	down
Pro-Lys	C11H21N3O3	1.8918	-0.3266	0.000792	down
3-Hydroxysebacic Acid	C10H18O5	1.8916	0.4574	0.000606	up
Pl	C28H46O10	1.8803	-0.2708	0.008032	down
Ecgonine	C9H15NO3	1.8722	0.2413	0.000832	up

Ecgonine Methyl Ester	C10H17NO3	1.8654	0.2268	0.000966	up
3,4-Dihydroxybenzenesulfonic Acid	C6H6O5S	1.8596	-0.1395	0.01439	down
Phenylalanyl-Alanine	C12H16N2O3	1.8545	-0.1937	0.001372	down
L-Pyridosine	C12H18N2O4	1.8516	-0.2256	0.001453	down
Dopamine	C8H11NO2	1.8508	0.2392	0.001066	up
Alpha-Methyltryptamine	C11H14N2	1.8455	-0.2164	0.001527	down
Dhv-Pge2	C22H34O5	1.8452	0.3301	0.001167	up
Cis-4-Hydroxycyclohexylacetic Acid	C8H14O3	1.8419	0.4386	0.001277	up
N-(1,3-Dihydroxy-3-Methylpentan-2-Yl)-2-Methylhexa-2,4-Dienamide	C13H23NO3	1.8417	0.3994	0.001236	up
Prima-1	C9H15NO3	1.8377	0.2521	0.001283	up
Glycerol 3-Phosphate	C3H9O6P	1.8287	0.2465	0.01789	up
2-N-Propyl-4-Oxopentanoic Acid	C8H14O3	1.8224	0.5404	0.001531	up
8-Nonenoylglycine	C11H19NO3	1.8209	0.3088	0.001555	up
1-(2-Methoxyphenyl)Ethanone	C9H10O2	1.8179	-0.1972	0.002677	down
Sterebin E	C20H34O4	1.8178	0.4929	0.000812	up
Proline Betaine	C7H13NO2	1.8171	0.1464	0.001901	up
Glycerol 2-Phosphate	C3H9O6P	1.8128	0.2357	0.01935	up
6-Octenoylcarnitine	C15H27NO4	1.8127	0.1878	0.001984	up
4-Amino-3-Hydroxybutanoylcarnitine	C11H22N2O5	1.8067	-0.6099	0.01149	down
1,2,4-Trideoxy-3-C-Methyl-4-{[(2E,4E)-2-Methyl-2,4-Hexadienoyl]Amino}Pentitol	C13H23NO3	1.797	0.5512	0.002367	up
N-(4-Oxopentyl)Acetamide	C7H13NO2	1.7961	0.1637	0.002392	up
3-Hydroxy-N-(2-Oxooxolan-3-Yl)Hexanamide	C10H17NO4	1.796	0.3693	0.002439	up
Glutamylleucylarginine	C17H32N6O6	1.7958	-0.2451	0.03424	down
3-Methylheptanedioylcarnitine	C15H27NO6	1.7917	0.1786	0.001122	up
Valtrate	C10H12O5	1.7898	-0.2117	0.01137	down
Tranexamic Acid	C8H15NO2	1.7881	0.1625	0.002165	up
Artemisin	C15H18O4	1.7778	-0.1957	0.0181	down
2-(S-Glutathionyl)Acetyl Glutathione	C22H34N6O13S2	1.7675	0.1619	0.01418	up
5-Methoxy Methylone	C12H15NO4	1.7673	0.5478	0.00289	up
1-(3-Hydroxy-7-Azaspiro[3.5]Nonan-7-Yl)-2-Methoxyethanone	C11H19NO3	1.7647	0.3331	0.00298	up
Dattelic Acid	C16H16O8	1.7591	0.1537	0.01598	up
Rhodamine 6G	C28H30N2O3	1.7349	-0.5075	0.00155	down
Trans-Zeatin	C10H13N5O	1.7335	0.2708	0.01904	up
Cer[Ns](D36:1)	C36H71NO3	1.7196	0.2944	0.01711	up
Gly-Gln	C7H13N3O4	1.7147	-0.3586	0.02697	down
Indolophenanthridine	C19H18N2	1.7029	-0.2798	0.002554	down
Trimethylamine N-Oxide	C3H9NO	1.7015	0.1441	0.003697	up
Amphotericin B	C47H73NO17	1.6913	-0.1629	0.02517	down
(2S)-2-Hydroxy-2-(Propan-2-Yl)Butanedioylcarnitine	C14H25NO7	1.6876	0.2705	0.002804	up

Crenolanib	C26H29N5O2	1.6757	-0.44	0.004176	down
Isoquercetin	C21H20O12	1.6681	0.8137	0.02464	up
1-(3-Hydroxy-7-Azaspiro[3.5]Nonan-7-Yl)-3-Methoxypropan-1-One	C12H21NO3	1.6665	0.6252	0.008288	up
6-(2-Aminopropyl)Indole	C11H14N2	1.6407	-0.4142	0.01358	down
Pc(O-16:0/0:0)	C24H52NO6P	1.6318	-0.162	0.005176	down
(3S)-3-Hydroxycyclocitral	C10H16O2	1.63	-0.1854	0.007642	down
(S)-A-Amino-2,5-Dihydro-5-Oxo-4-Isoxazolepropanoic Acid N2-Glucoside	C12H18N2O9	1.6237	0.1376	0.0223	no change
6-Ketoprostaglandin F1Alpha	C20H34O6	1.6236	-0.2416	0.01236	down
N-Methyl-2-(4'-Methylaminophenyl)-6-Hydroxybenzothiazole	C15H16N2OS	1.6194	-0.157	0.03662	down
2-(4-Methoxyphenyl)Ethyl Hydrogen Sulfate	C9H12O5S	1.6174	-0.2262	0.03517	down
2,6-Dihydroxybenzoic Acid	C7H6O4	1.6099	0.1763	0.02907	up
Neuroprotectin D1	C22H32O4	1.6085	0.4218	0.005344	up
N-Methyl-2,3-Dihydro-1H-Inden-2-Amine	C10H13N	1.6017	-0.3123	0.01297	down
4-Aminohippuric Acid	C9H10N2O3	1.555	0.182	0.03786	up
Delapril Diacid	C24H28N2O5	1.5486	-0.2855	0.01414	down
Prolyl-Gamma-Glutamate	C10H17N3O4	1.5344	-0.1544	0.02954	down
2,8-Quinolinediol 2-Sulfate	C9H7NO5S	1.5289	0.4088	0.02779	up
Lys-Gly-Oh	C13H17N3O6	1.5147	-0.1494	0.03038	down
Gly-Pro	C7H12N2O3	1.4931	-0.2765	0.02371	down
Kaempferol 3-O-Galactoside	C21H20O11	1.4849	0.1765	0.03998	up
Kinetin	C10H9N5O	1.4707	-0.1743	0.0224	down
Glu-Glu-Tyr	C19H25N3O9	1.4149	-0.212	0.02624	down
Dalbergioidin	C15H12O6	1.3769	0.1575	0.04983	up
3-(1,2,3,4-Tetrahydroisoquinolin-2-Yl)-2-(2-Thienylcarbonyl)Acrylonitrile	C17H14N2OS	1.3718	-0.1935	0.02983	down
Tripeptide	C24H35N7O8	1.3513	-0.2732	0.04041	down

Table S4

Effects of Roughage Sources on Growth Performance in Dumont Sheep and Mongolian Sheep

Items	Treatments[1]				SEM	*p*-value[2]			
	Dumont Sheep		Mongolian Sheep			T	B	R	B×R
	AH	CS	AH	CS					
IBW, kg	20.25	20.2	19.7	19.47	0.15	0.186	0.038	0.628	0.754
FBW, kg	39.47	37.11	38.02	35.58	0.33	<0.001	<0.001	<0.001	0.914
ADG, g/d	213.58	187.84	203.50	178.98	3.65	<0.001	0.074	<0.001	0.905
ADFI, kg/d	1.68	1.56	1.60	1.48	0.02	<0.001	<0.001	<0.001	0.948
FCR, g/g	7.88	8.35	7.89	8.32	0.13	0.427	0.9723	0.104	0.930

IBW, initial body weight; FBW, final body weight; ADG, average daily gain; ADFI, average daily feed intake; FCR, feed conversion ratio.

[1] AH: alfalfa hay diet, CS: corn stalk diet. 2 T = different treatments; B = breed (Dumont sheep × Mongolian sheep); R = roughage (AH × CS); B × R = breed × roughage, breed by roughage interaction.

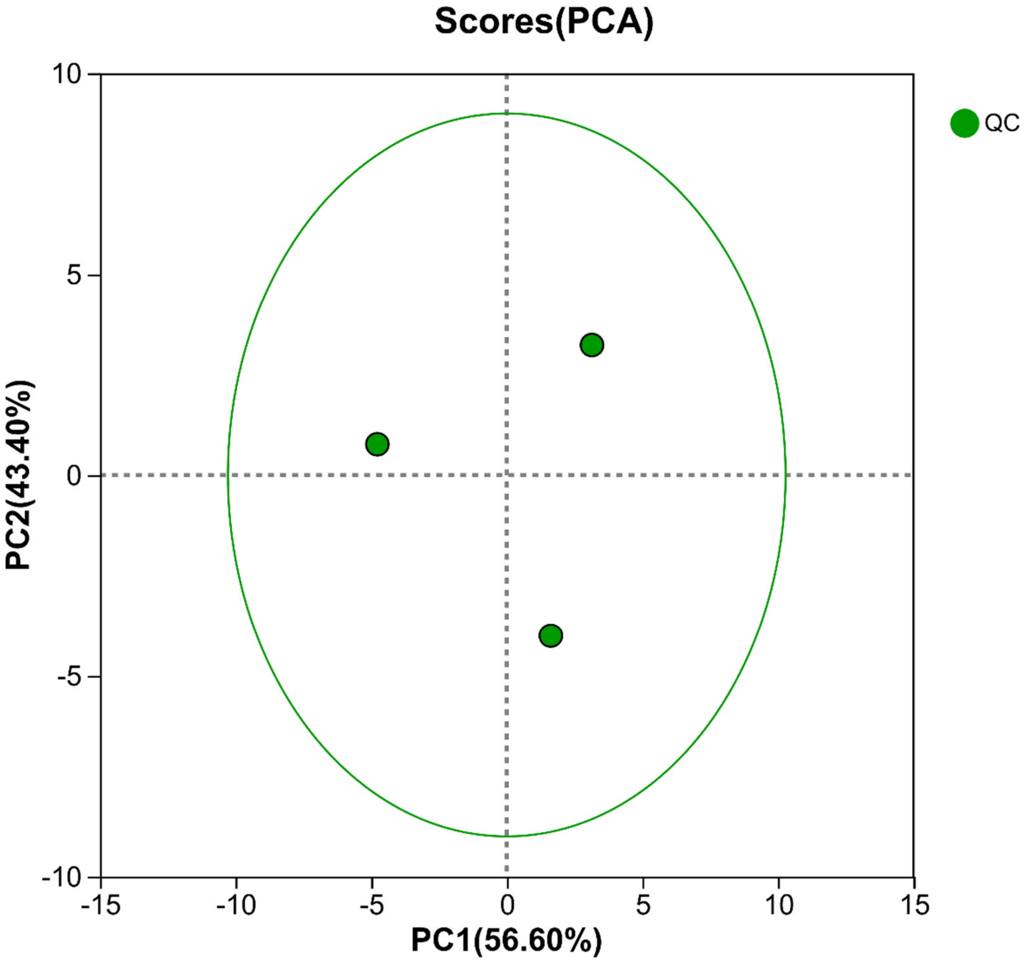

Figure S1 PAC Analysis of QC Samples

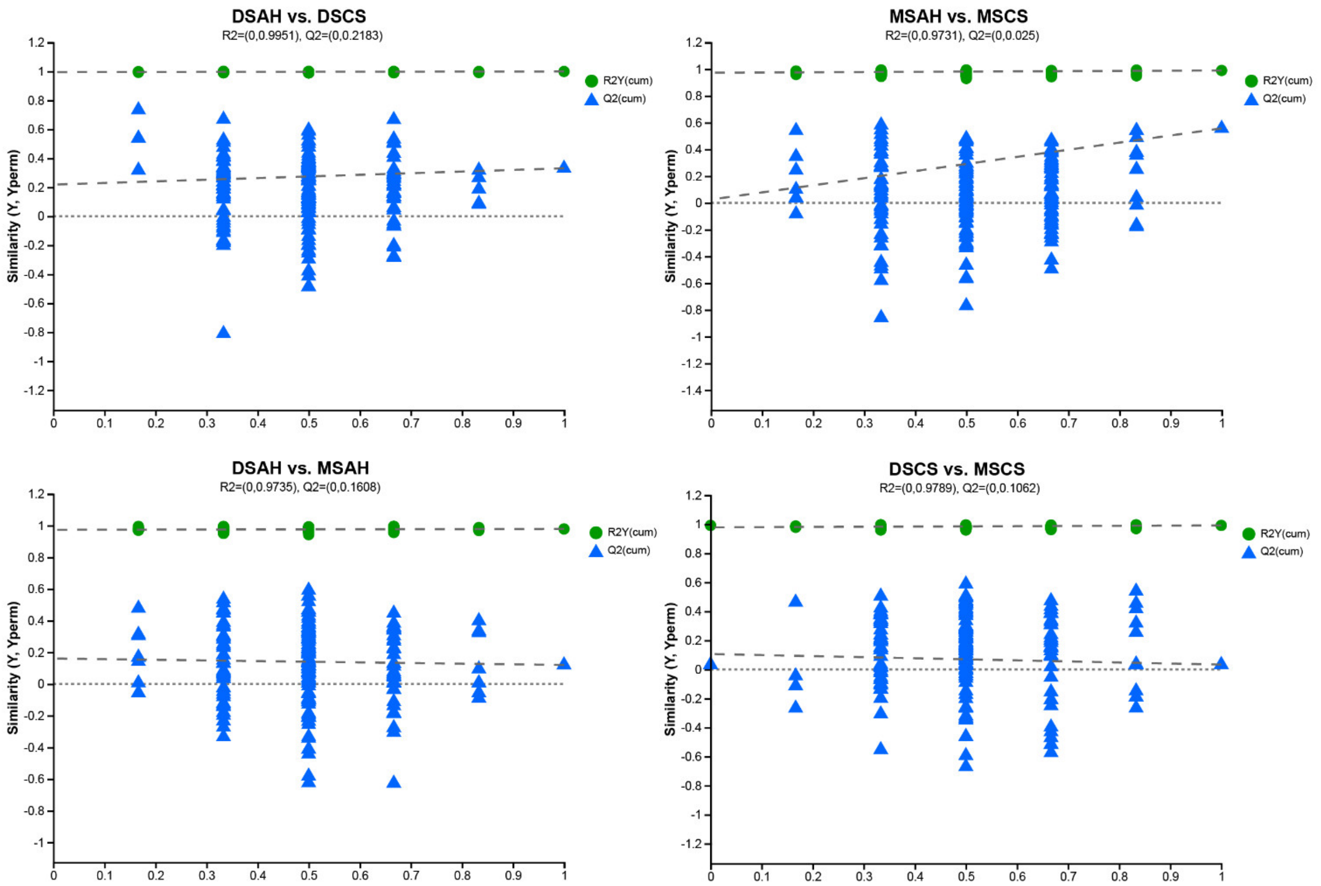

Figure S2 Plot of OPLS-DA permutation of the DSAH vs. DSCS; MSAH vs. MSCS; DSAH vs. MSAH; DSCS vs. MSCS.
